# *Lacticaseibacillus rhamnosus*: A Suitable Candidate for the Construction of Novel Bioengineered Probiotic Strains for Targeted Pathogen Control

**DOI:** 10.3390/foods11060785

**Published:** 2022-03-08

**Authors:** Moloko G. Mathipa-Mdakane, Mapitsi S. Thantsha

**Affiliations:** Department of Biochemistry, Genetics and Microbiology, University of Pretoria, Pretoria 0002, South Africa; molokog.mathipa@gmail.com

**Keywords:** *Lacticaseibacillus rhamnosus*, probiotics, pathobiotechnology, recombinant probiotics, pathogen

## Abstract

Probiotics, with their associated beneficial effects, have gained popularity for the control of foodborne pathogens. Various sources are explored with the intent to isolate novel robust probiotic strains with a broad range of health benefits due to, among other mechanisms, the production of an array of antimicrobial compounds. One of the shortcomings of these wild-type probiotics is their non-specificity. A pursuit to circumvent this limitation led to the advent of the field of pathobiotechnology. In this discipline, specific pathogen gene(s) are cloned and expressed into a given probiotic to yield a novel pathogen-specific strain. The resultant recombinant probiotic strain will exhibit enhanced species-specific inhibition of the pathogen and its associated infection. Such probiotics are also used as vehicles to deliver therapeutic agents. As fascinating as this approach is, coupled with the availability of numerous probiotics, it brings a challenge with regard to deciding which of the probiotics to use. Nonetheless, it is indisputable that an ideal candidate must fulfil the probiotic selection criteria. This review aims to show how *Lacticaseibacillus rhamnosus*, a clinically best-studied probiotic, presents as such a candidate. The objective is to spark researchers’ interest to conduct further probiotic-engineering studies using *L*. *rhamnosus*, with prospects for the successful development of novel probiotic strains with enhanced beneficial attributes.

## 1. Introduction

Probiotics, defined as “live microorganisms which when administered in adequate amounts confer a health benefit on the host” [1], are added into functional products for the improvement of human and animal health [2]. Traditionally, probiotic strains belong to *Lactobacillus* and *Bifidobacterium* species [2], but have also recently included other lactic acid bacteria such as *Streptococcus*, *Leuconostoc*, *Pediococcus*, *Enterococcus*, *Propionibacterium*, and *Streptococcus*, and some other microorganisms, such as *Saccharomyces* and *Bacillus* [2,3]. Evaluations of these microbes found no major safety concerns for their application in foods and dietary supplements [4]. The number and types of probiotic microorganisms are likely to increase as researchers continuously explore various sources to isolate novel probiotic strains with superior probiotic attributes, in order to meet the probiotic market demand [2]. Among the various *Lactobacillus* strains regarded as probiotics, *Lacticaseibacillus rhamnosus*, formerly known as *Lactobacillus rhamnosus* [5], has been widely studied. Selected strains belonging to *L. rhamnosus* species are extensively used as probiotics in food formulations, health, and functional foods [6]. The *L. rhamnosus* strain GG (Gorbach–Goldin), one of the most well-documented probiotic microorganisms, originally isolated from faecal samples of a healthy human adult, has been identified as a potential probiotic strain [7]. It has been deemed a probiotic because of its resistance to acid and bile as well as its good growth characteristics that allow it to survive and persist within the gastrointestinal tract [8]. It has also been reported to be highly resistant to technological processes and has a great adhesion capacity to the intestinal epithelial layer to subsequently inhibit the growth and adherence of several pathogens [9,10].

*Lacticaseibacillus rhamnosus* can survive and thrive through the gastrointestinal tract while adhering to the intestinal epithelial cells. This strain has been displayed as an excellent mucus-adhering *Lactobacillus* strain when compared to related strains such as *Lactobacillus johnsonii* LJ1 and *Lacticaseibacillus casei* Shirota [11]. Additionally, Martín et al. [12] reported that it is able to form biofilms, enhancing its ability to protect and strengthen the cytoskeleton integrity to inhibit pathogen colonisation. Segers and Lebeer [13] stated that LGG’s strong adhesive capacity and efficacy against gastrointestinal (GI) pathogens have been documented in vitro and also in vivo in humans. A corroborative study by Vélez et al. [14] reported that *L. rhamnosus* GG adheres very well to the intestinal mucosa. To show the efficacy of this probiotic against pathogens, Marianelli et al. [15] reported that *L. rhamnosus* GG was able to inhibit *Salmonella enterica* subsp. enterica serovar Typhimurium 1344 in vitro. Later, Zhang et al. [16] also showed that *L. rhamnosus* was able to inhibit *Shigella sonnei* in vitro.

Amongst other benefits, *L. rhamnosus* has been well documented for its clinical benefits. In order for any microorganism to have clinical effects, it has to adhere to and colonise the GIT, and *L. rhamnosus* has been shown to possess these traits in different studies [13]. Different studies have reported on the use of *L. rhamnosus* GG for the prevention and treatment of gastrointestinal infections and diarrhoea in children [17,18]. Hojsak et al. [19] reported that when administered to children, it was able to reduce the duration of diarrhoea and the risk of acquiring nosocomial gastrointestinal infections. Horvath et al. [20] reported that the administration of this probiotic reduced for its clinical benefits, the pain frequency and intensity of abdominal pain-related disorders in patients with irritable bowel syndrome (IBS). When it comes to pathogens, administration of *L. rhamnosus* GG was reported to prevent enteric colonisation by *Candida* species [21,22] and to treat recurrent *Clostridium difficile* induced colitis in children [23]. In a recent study, Slykerman et al. [24] evaluated the effect of *L. rhamnosus* HN001 (HN001) on symptoms of maternal depression and anxiety during the postpartum period. Their results showed significantly lower depression and anxiety scores in women who received this strain.

*L*. *rhamnosus* is a well-studied lactic acid bacterium that possesses desirable features of conventional probiotic strains. In this study, different databases (Science Direct, Google Scholar, and PubMed) were searched for published scientific original and review articles on *L. rhamnosus*. The following keywords were used to search for articles: probiotics, probiotic properties, probiotic criteria, *L. rhamnosus*, pathobiotechnology, probiotic engineering, and recombinant probiotics. The articles most relevant to the study are summarised and presented here. According to the information gathered, probiotic engineering is a promising strategy for the development of robust probiotic strains. However, such studies, specifically those aiming to bioengineer *L. rhamnosus* for specific applications, including targeted pathogen control, are scarce despite its many proven desirable probiotic attributes.

The rationale for this review is therefore to present evidence in favour (to vouch for) of *L. rhamnosus* as a good choice probiotic candidate for more probiotic engineering studies, with great prospects to deliver dual benefits, that is, conventional probiotic beneficial effects, together with the functional attribute(s) of the gene(s) introduced into and expressed by the bioengineered strain, among others, enhanced targeted control of a specific pathogen, due to the ability of the bioengineered strain to compete for and bind to the same receptor(s) as the targeted pathogen. It highlights the desirable attributes possessed by *L. rhamnosus*, and then summarises the probiotic engineering studies that have been conducted on *L. rhamnosus* to date, their successes and limitations, as well as the potential prospects for its applications in further probiotic engineering studies.

## 2. Desirable Properties of Probiotics

Consumers are in need of food that can be beneficial to them in one way or another. Taking this into consideration, different markets are exploring products with probiotic microorganisms in an effort to improve the properties of indigenous microflora [25]. Probiotics, in their definition, are beneficial microorganisms which exert a positive effect on the host, improving the intestinal microbiota [26]. However, it is still crucial to report that all characteristics attributed to probiotics are, in general, strain-specific, and individual strains have to be tested for each property [27].

**Figure 1:**
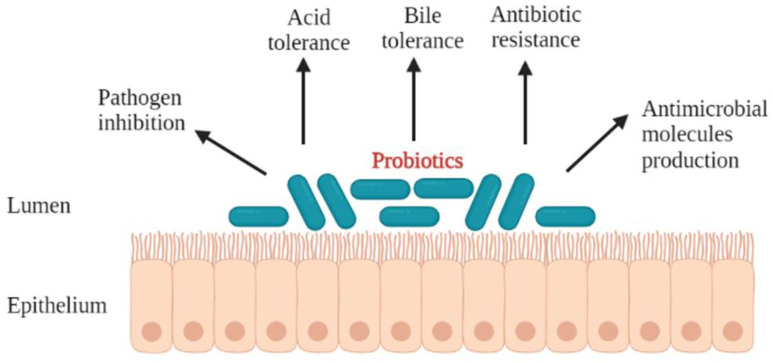
Desirable properties of the probiotic microorganisms.

The criteria used to select for probiotics have been reported by different researchers [27]. In order for a microorganism to be used as a probiotic, it should preferably be of human origin, possess a generally regarded as safe (GRAS) status, and be able to survive through the gastrointestinal tract [28]. The most studied probiotics are the lactic acid bacteria, particularly *Lactobacillus* and *Bifidobacterium*. This is attributed to the fact that most *Lactobacillus* species are normal inhabitants of the human and animal intestine, and their presence is important for the maintenance of the intestinal microbial ecosystem [29]. All probiotics taken orally travel via the mouth and transit through the gastrointestinal tract, a journey that exposes them to a myriad of successive stress factors that negatively influence their survival [25]. In order for a probiotic to eventually colonise and exert beneficial health effects in the host, it has to exhibit most, if not all, of the desirable properties (Figure 1). These include, but are not limited to, the ability to: tolerate acid and bile salts [2,30,31,32,33], produce a variety of antimicrobial compounds [2,34], inhibit pathogens, and colonise the gastrointestinal tract of the host [2,32,33,35]. In addition to these, the probiotic should not harbour transferable antibiotic resistance genes [36,37].

The viability of a probiotic in the human upper GI tract is influenced by several factors [38] including low acidity of the stomach [39,40]. These stress factors have been reported to destroy bacterial cell membranes, thereby decreasing their viability and ability to permeate through different cells [41]. It is therefore crucial that all probiotics are able to show a tolerance to acid to assure their survival in acidic conditions [42]. In an effort to select strains of probiotic interest, in vitro methods aiming to ascertain their survival through the upper gastrointestinal tract and arrive alive at their site of action are used. Another property required by probiotics for their survival in the small intestine is tolerance to bile [2,43]. The presence of bile salts presents a barrier to the survival of ingested microorganisms during digestion [32]. Depending on its concentration, bile can inhibit the growth of bacteria [44]. Therefore, the ability of probiotics to tolerate bile has been analysed by measuring the bacterial growth in the presence of bile salts [45,46,47]. The probiotics’ tolerance to bile means that they will be able to reach the large intestine, where they exert their beneficial effects.

**Figure 2:**
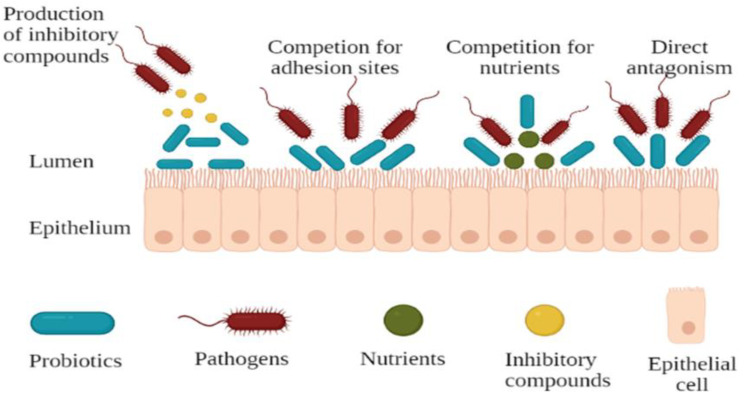
Mechanisms of pathogen inhibition by probiotics.

The ability to survive through the GIT alone does not make a microorganism a probiotic. Potential probiotic strains should have the ability to adhere to mucosal surfaces of the intestinal tract, have desirable antibiotic resistance and sensitivity patterns, be antagonistic toward potentially pathogenic microorganisms, and have metabolic activities beneficial to the well-being of the host [28]. Previous research has reported that most lactic acid bacteria (LAB) produce a variety of antimicrobial compounds when in different niches such as the gastrointestinal tract [34,48]. Probiotic microorganisms should be able to inhibit pathogens. This inhibition is achieved via different mechanisms (Figure 2) including the inhibition of pathogens through competitive exclusion, production of specific antimicrobials, and stimulation of barrier function and metabolic function [49]. Additionally, they have been shown to possess inhibitory activity towards the multiplication of enteropathogens and show a competitive ability through the production of several antimicrobial compounds [50]. Probiotics are able to inhibit pathogens through adhesion and colonisation in the gastrointestinal tract of the host [35]. These properties have been investigated in various bacterial species in food and in the intestinal tract over the years to obtain adhesive probiotic bacteria [51].

There has been an increased interest in studying the capability of probiotics for the prevention and treatment of different infections. This has been attributed to their ability to inhibit pathogens from binding to epithelial cells and has been demonstrated in models of the gut epithelium [52]. One mechanism that probiotics use to inhibit pathogen binding is through competitive exclusion, which has been previously defined as an extensive phenomenon in nature that includes the competition for nutrients [53,54] and for physical space [55]. Previous studies have investigated and showed that competitive exclusion was a major mechanism of growth inhibition of bacterial [56] and fungal growth [57]. Forestier et al. [9] and Pitino et al. [58] stated that the adhesion capability of the probiotic organism to the mucosal cells of the gut is considered important for competitive exclusion of enteropathogens and immuno-modulation of the host, respectively. Understanding this competition mechanism, probiotic bacteria can thus be utilised to play a role on health, such as for treatment of diseases of the urogenital tract and oral cavity [59].

**Figure 3:**
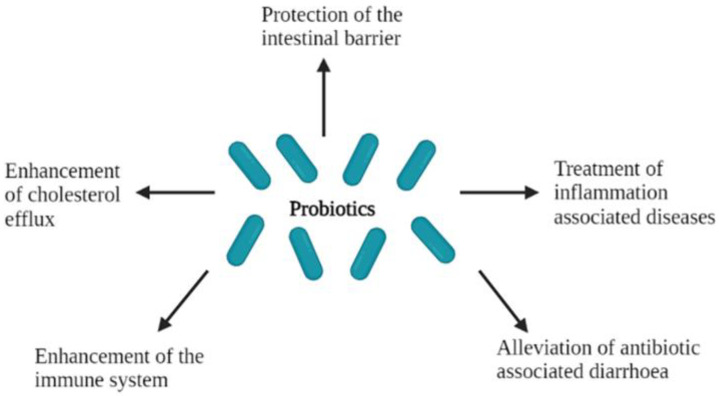
Health effects of probiotics in the host.

The ability of probiotics to play a role and confer health benefits to the host has been well studied and consequently documented (Figure 3). Amongst the different applications that probiotics are used for, they have been reported to protect the host’s intestinal barrier [60], enhance the immune system [2,61,62] and the cholesterol efflux [2,63,64], treat inflammation associated diseases [65,66] and alleviate antibiotic-associated diarrhoea [67].

One of the most important aspects when it comes to the functioning probiotic bacteria is their ability to protect the host’s gastrointestinal microenvironment from invading pathogens. They achieve this through the maintenance of both the well-being of commensal bacteria and integrity of the intestinal barrier [60]. The ability of probiotics to adhere to the intestinal cells will offer enhanced effects such as exerting these beneficial effects while competing for space with other microorganisms. Previous research has reported that the ability of a probiotic to adhere to intestinal cells is a prerequisite to colonise [68], stimulate the immune system [69], and show antagonistic activity against enteropathogens [59,70]. Additionally, the ability of the probiotic to adhere to and colonise mucosal surfaces [71] provides an opportunity for the probiotic to influence the host [72].

There have been growing reports highlighting the significance of probiotics in the gastrointestinal tract for the attenuation of intestinal inflammatory diseases [73]. However, it is also worth noting that there is an increase in reports about probiotics influencing the immune responses outside the gastrointestinal tract, including the respiratory mucosa [61,62]. In immunocompromised malnourished hosts, LAB have been reported to be beneficial through their ability to modulate the immune system (immunobiotics), thus representing an attractive, safer way to regulate and enhance the immune function [62]. The effect of different immunobiotics in the prevention of opportunistic infections in the immunocompromised were evaluated, and the results showed that probiotic-based diets offered beneficial effects as they significantly accelerate the recovery of immune systems, thus improving resistance against pathogens [61,74]. Lactobacilli species have been reported to exert different properties upon interaction with the mammalian immune system [75,76] including the production of immuno-stimulatory molecules [77]. Bermudez-Brito et al. [78] reported that there was accumulating evidence showing that probiotics are capable of exerting immunomodulatory effects.

## 3. How Well Does *L. rhamnosus* Fulfil the Probiotic Selection Criteria?

In order for *L. rhamnosus* to be referred to as a probiotic, it needs to fulfil all the aspects listed under properties of probiotics. Probiotic benefits of *L. rhamnosus* GG have been well studied and reported by various researchers; thus, it has been extensively used in a variety of functional foods [79,80].

### 3.1. Ability to Endure Gastrointestinal Stresses, Acid and Bile Tolerance

For probiotics to confer their beneficial effects on the host, they are ingested with different vehicles and travel through the gastrointestinal tract (GIT). It is during this transit that they are exposed to successive stress factors, including the stomach acid and the secreted bile, that further influence their survival [81]. It has been reiterated that for *L. rhamnosus* to obtain its probiotic status, it needs to preserve its viability under the harsh gastrointestinal conditions [82,83]. To further enhance the survival of *L. rhamnosus* in the GIT, Succi et al. [25] investigated the effects that prebiotics could have. They found when pre-cultivated with some prebiotics, *L. rhamnosus* exhibits enhanced resistance and consequently viability under simulated GI transit.

Previous studies have investigated the ability of *L. rhamnosus* as a probiotic candidate; this was achieved through checking the different properties. A study by Succi et al. [84] investigated the capability of *L. rhamnosus* strains isolated at the end of the ripening of Parmigiano Reggiano cheese to survive at low pH and in the presence of bile salts. They reported that the strains were able to grow and survive at lower pH and in the presence of bile concentrations as high as 2%. In a different study by [85], they isolated different *L. rhamnosus* strains from different sources and screened them for tolerance to acid and bile salts, among other properties. They found that the strains showed tolerance to high acidity and bile salts. One of the most used probiotic strains, *L. rhamnosus* GG, has been reported to be resistant to acid and bile [13]. These results in turn make *L. rhamnosus* strains candidates for probiotics.

The tolerance of microorganisms to bile stress is most crucial as it has been reported by Mirlohi et al. [86] that bile stress can have detrimental effects on microbes. Pace et al. [87] reported that all probiotics should be able to survive exposure to the bile salts in the small intestine to retain their viability in the large intestines. Lebeer et al. [77] stated that when bacteria are exposed to bile, they modify their cell envelope properties such as the cell membrane fatty acid composition, peptidoglycan composition, and membrane charge, avoiding the deleterious effects. In an effort to understand the bile-tolerance mechanism in *L. rhamnosus* GG, Koskenniemi et al. [83] used gene expression to investigate its cellular response toward bile. Their results showed that when grown in 0.2% ox gall, *L. rhamnosus* GG can express stress responses that are likely to strengthen the cell envelope against bile-induced stress. A previous study by Reale et al. [85] investigated the ability of different *L. rhamnosus* strains to tolerate bile salts; they reported that the strains that were screened showed a good ability to grow in the presence of 1.5% bile salts. These results were in agreement with those from a study by Shi et al. [88], who exposed *L. rhamnosus* strains to different bile salt concentrations (0.3%, 0.5%, and 1%) and reported that there was a stable maintenance of cell numbers.

### 3.2. Antipathogenicity Effects

Amongst other ways, Rolfe [89] stated that probiotics provide protection to the host through the inhibition of the colonisation of pathogens and growth thereof. The inhibition of pathogens by *L. rhamnosus* in the gastrointestinal tract was attributed to its ability to colonise the epithelial cells, strengthening the intestinal barrier and thus modulating the innate and adaptive immune responses [90,91]. Previous studies by De Keersmaecker et al. [92], Beristain-Bauza [93], and de Alcântara et al. [94] reported the ability of *L. rhamnosus* to reduce the viability of *Salmonella enterica* serovar Typhimurium, *Staphylococcus aureus*, and *Pseudomonas fluorescens*, respectively, in vitro. In an effort to understand this better, Vesterlund et al. [95] showed that *L. rhamnosus* GG was able to reduce the adhesion of *Staphylococcus aureus* to intestinal cells by as much as 44%, ultimately lowering the infection rate. In a different study, Wong et al. [96] investigated the ability of *L. rhamnosus* GG to inhibit *Streptococcus pneumonia*, and reported that there was an inhibition of pathogen colonisation, suggesting suitability of *L. rhamnosus* for clinical use. Later, Mohammedsaeed et al. [97] investigated whether *L. rhamnosus* GG could protect keratinocytes from the pathogenic effects of *S. aureus*. They reported that *L. rhamnosus* GG protected keratinocytes using multiple mechanisms including inhibition of pathogen growth.

Having stated that the performance of probiotics can be strain- and pathogen-specific, Johnson-Henry et al. [98] investigated the ability of *L. rhamnosus* GG in the protection of the epithelial cell barrier after being challenged with enteric pathogen *E. coli* O157:H7. They reported that their co-incubation resulted in a significant reduction in *E. coli*’s capability to change cell morphology and manipulate the distribution and expression of tight junction proteins. Preceding this, the study by Ephraim et al. [99] investigated the anti-cytotoxic effects of *L. rhamnosus* GG (LGG) against *C. difficile* toxins; they reported that LGG inhibited the growth of and protected the cells from induced pathogen cytotoxicity in vitro.

Another mechanism that probiotics use to inhibit pathogens is through the production of different antimicrobial substances. A few studies have demonstrated that some *L. rhamnosus* strains are capable of secreting antimicrobial substances that can inhibit other bacteria [16,100]. Wong et al. [96] investigated the inhibition of *Streptococcus pneumoniae* colonisation and found that *L. rhamnosus* inhibited the colonisation of all isolates in vitro. This inhibition was attributed to competition for binding to molecules such as fibronectin and collagen, as both *S. pneumoniae* and *Lactobacillus* species have been shown to bind to these molecules [101]. Jeong and Moon [102] isolated a bacteriocin (rhamnocin 519) from *L. rhamnosus*, which exhibited antimicrobial activity towards *L. monocytogenes* and *S. aureus*. Allonsius et al. [22] looked at the inhibition of *Candida albicans* by *L. rhamnosus* GG. They found that the antimicrobial chitinase was responsible for the inhibition, and thus can be used in better strain selection in *Candida* management strategies. Biswas et al. [103] isolated an *L. rhamnosus* LRB strain from a healthy baby-tooth and assessed its ability to inhibit streptococci. They reported that the LRB strain secreted antimicrobial agents that inhibited a variety of streptococci including *Streptococcus mutans*. In yet another study, *L*. *rhamnosus* isolated from breastmilk was shown to produce a biosurfactant with antibiofilm properties against *Bacillus subtilis*, *Pseudomonas aeruginosa*, *S*. *aureus*, and *E*. *coli*. This glycolipid biosurfactant reportedly inhibits the attachment of the pathogens to the surfaces, consequently disrupting biofilm formation through the alteration of the integrity and viability of the bacteria within the biofilms [104].

To add to this, Makras et al. [105] stated that *L. rhamnosus* is a lactic-acid-producing bacterium; therefore, it is possible that some of the antimicrobial activity is due to lactic acid. This notion was further supported by De Keersmaecker et al. [92], who investigated and surveyed different antimicrobial compounds produced by *L. rhamnosus* against *Salmonella*; they found that the strong antimicrobial activity was mediated by lactic acid. In addition to that, Hutt et al. [106] and later Zhang et al. [16] determined the concentration of lactic acid produced in the culture medium, and reported that it is dependent on the growth conditions of *L. rhamnosus*. This production of antimicrobial substances by *L. rhamnosus* has been proven to be effective in the inhibition of different pathogens.

In addition to all these in vitro studies, other researchers looked at the ability of *L. rhamnosus* to inhibit pathogens in vivo. A previous study by Tytgat et al. [107] investigated a mechanism of competitive exclusion of *Enterococcus faecium* by *L. rhamnosus* GG. In this work, the authors demonstrated how the possession of similar pili structures made it possible for *L. rhamnosus* to inhibit the colonisation of *E. faecium*. Succeeding that, Zhou and Zhang [108] investigated the antibacterial effects of bacteriocins obtained from *L. rhamnosus* on a rabbit model of *S. aureus* infection following knee replacement surgery. Their results indicated that bacteriocins are a potential agent for the prevention of orthopaedic postoperative infections. Naik et al. [109] investigated the ability of probiotic *L. rhamnosus* GG in protecting neonatal mice against *Salmonella* infection. In their results, they showed that pre-treatment of the mice with the probiotic resulted in enhanced anti-inflammatory cytokine expression and increased gut barrier function, rescuing 80% of the *Salmonella*-infected mice. 

### 3.3. Protection of the Epithelial Barrier

The adherence capabilities of *L. rhamnosus* to the epithelial layer have been reported to protect and strengthen the epithelial barrier, thus enhancing the inhibition of pathogenic microorganisms [13]. Taking this into consideration, *L. rhamnosus* GG represents one of the clinically best-studied probiotic organisms and that is widely used in the prevention and treatment of different gastrointestinal infections [110,111] due to its exhibition of protective effects on the integrity and function of the intestinal barrier [112].

Due to the popular use of *L. rhamnosus* GG, different studies have reported on the ability of *L. rhamnosus* to adhere and colonise epithelial cells both in vitro and in vivo [51]. A previous study by Van Tassell and Miller [113] stated that adhesion to the intestinal mucosal surface is an important prerequisite for colonisation of *Lactobacillus*, providing them a competitive advantage. During surface adhesion, *L. rhamnosus* GG uses the pilin SpaC subunit to bind to the human mucin and intestinal epithelial cells [114] and MabA for adhesion to epithelial cells and biofilm formation [115]. Shi et al. [88] isolated *L. rhamnosus* strains and checked their ability to adhere to the extracellular matrix, amongst other criteria. They reported that different strains showed binding to both fibronectin and laminin.

A previous study by Johnson-Henry et al. [98] investigated the capability of *L. rhamnosus* GG to protect the epithelial cell barrier in response to challenge with the enteric pathogen *E. coli* O157:H7 and to delineate the mechanistic aspects by which *L. rhamnosus* GG exerts its effects. Their results indicated that *L. rhamnosus* GG protected the epithelial barrier and prevented it from the cellular morphological changes usually induced by *E. coli* O157:H7 injury. Huang et al. [116] examined the suitability of *L. rhamnosus* as a prophylactic treatment in neonatal sepsis and meningitis caused by *E. coli* K1 in a murine model. They reported a strong *E*. *coli* K1 suppression, indicated by a significant reduction in its levels of intestinal colonisation in neonate rats treated with the probiotic. Recently, Han et al. [117] studied the protective effects of *L*. *rhamnosus* GG on epithelial barrier function using human intestinal epithelial cultures (enteroids and colonoids), and they reported that pre-treatment of the cells with LGG prevented paracellular permeability, implying that it eliminated epithelial barrier dysfunction.

### 3.4. Enhancement of Immune Response

With more bacteria reported to exert immunomodulatory effects, *L. rhamnosus* has been reported to protect the intestinal epithelial cells from apoptosis, to promote its own proliferation in vitro [111,118], attributed to the immunomodulatory effects of the pili structure [13]. Salva et al. [74] demonstrated that *L. rhamnosus* CRL1505 increases resistance against pneumococcal infection in immunocompetent mice. Studies by Alvarez et al. [61] and Villena et al. [62] stated that *L. rhamnosus* CRL1505 has the capacity to impact the innate immune cells of different hosts and thus offer a protective effect. Herrera et al. [119] concluded that the administration of *L. rhamnosus* CRL1505 normalised the systemic and respiratory innate immune responses in malnourished mice, after they observed improvements in neutrophil recruitment, higher phagocytic activity, and increased resistance against pneumococcal infection. In a different study investigating the effect of *L. rhamnosus* CRL1505 on the respiratory immunity against *Streptococcus pneumoniae*, it resulted in the absence of pneumococci in the lungs or blood samples, demonstrating it as an alternative mucosal immunomodulator in immunocompromised hosts. Furthermore, when Villena et al. [120] investigated the mechanisms involved in the immunomodulatory effect of the *L. rhamnosus* CRL1505 strain in viral infections, their results showed that it offered a potential to overcome the viral diarrhoea episodes as it was able to modulate the innate immunity and induced the production of antiviral type I IFNs, IFN-γ, and regulatory IL-10.

In an in vitro study with Caco-2 cells, Lebeer et al. [121] demonstrated that SpaCBA pili were key for the adhesion ability of *L. rhamnosus* to intestinal epithelial cells and that this adherence was necessary for its immunomodulatory activity. In addition, Vargas García et al. [122] showed that the *L. rhamnosus* SpaCBA pili also played an important role in its adherence to macrophages, and that this interaction promoted anti-inflammatory effects through the induction of IL-10 mRNA and reduction of IL-6 mRNA in murine macrophages. Kim et al. [123] investigated the effects of two strains of *L. rhamnosus*, *L. rhamnosus* GG and -GR-1, on modulating the production of tumour necrosis factor (TNF) in human monocytic cell line THP-1 and mouse macrophages. They found that these strains triggered anti-inflammatory effects, thereby suppressing TNF production in macrophages. In another study on macrophages, Bleau et al. [124] investigated immunosuppressive properties of the exopolysaccharide (EPS) from high-EPS producer *L. rhamnosus* RW-9595M, on inflammatory cytokines produced by macrophages. They reported that the strain induced immunosuppression through the production of macrophagic anti-inflammatory IL-10. Thus, the ability of *L. rhamnosus* to produce immunomodulatory molecules offers them an advantage in conferring beneficial effects to the host.

A previous study by Banna et al. [125] stated that *L. rhamnosus* possesses immunomodulatory activity and has been extensively used for the treatment of various diseases in vivo. Segers and Lebeer [13] attributed the immunomodulatory activity to the structural components or bioactive compounds, such as the immunomodulatory effects of the pili structure of *L. rhamnosus*. Mahnet et al. [126] investigated the immunomodulatory effect of *L. rhamnosus* GG on Swiss albino mice, and they reported that it stimulated the total humoral immune response. Goyal and Shukla [127] showed that oral administration of *L. rhamnosus* GG in *Giardia*-infected mice modulated both humoral and cellular immune systems, suggesting that it could be used as a bacteriotherapy for *Giardia* infections. In the same year, Harb et al. [128] assessed the immunomodulating effects of viable *L. rhamnosus* GG and its derived soluble mediators in vivo. Their results showed that the probiotic exerted immunomodulatory activities, and its administration in neonates may provide an alternative in reducing allergic inflammatory responses. In a different study, Dimitrijevic et al. [129] reported an enhanced innate immunity due to the administration of *L. rhamnosus* LA68 to healthy C57BL/6 mice with no underlying pathological condition. Saliganti et al. [130] investigated the effects of the consumption of *L. rhamnosus*-containing fermented milk on the development of immune systems of newborn babies. They reported that this milk had beneficial effects on the development of newborns’ immune systems with subsequent restoration of Th1/Th2 homeostasis.

## 4. Clinical Suitability: The Use of *L. rhamnosus* for Treatment of Disease

In addition to having the probiotic status, *L. rhamnosus* has demonstrated the ability to be a clinical bacterial strain and has thus been extensively utilised in a variety of commercially available probiotic products [131]. The beneficial effects of this strain have been studied extensively in the laboratory as well as in clinical trials and used in human intervention studies [13].

### 4.1. Alleviation of Antibiotic-Associated Diarrhoea

Antibiotics have always been widely prescribed for the treatment of most bacterial infections; however, there has been increasing caution when it comes to their use due to development of antibiotic resistance in bacteria. They have also been reported to result in different infections including antibiotic-associated diarrhoea (AAD) in paediatric patients [132]. Since it is understood that AAD is caused by dysbiosis that it is triggered by the administration of antibiotics, an alternative treatment would be more appropriate if it could restore the gastrointestinal microflora. Probiotics have been reported to offer beneficial effects to the host and are able to modulate and normalise the unbalanced indigenous gastrointestinal microbiota [67]. *Lacticaseibacillus rhamnosus* is reportedly capable of reducing the duration and severity of antibiotic-associated diarrhoea [133,134]. Arvola et al. [135] assessed the ability of *L. rhamnosus* GG as a potential preventive option for antibiotic-associated diarrhoea, and they reported that it was effective in preventing diarrhoea in children. Similarly, Canani et al. [136], Grandy et al. [137], and Hojsak et al. [138] also reported the efficacy of *L. rhamnosus* GG for the treatment of acute diarrhoea in children using in vivo studies. Amongst probiotics that they studied, Wolvers et al. [139] also reported that *L. rhamnosus* GG (LGG) showed potential in the treatment and prevention of gastrointestinal complaints.

Szajewska et al. [18] investigated the efficacy of *L. rhamnosus* GG in the prevention of healthcare-associated diarrhoea, and they found that it lowered the diarrhoea rates and caused no harm in any of the trials. Recently, Goldenberg et al. [140] evaluated and compared the effects of different probiotics in preventing AAD; they reported that *L. rhamnosus* GG was one of the most appropriate probiotics. In another study, Szajewska and Kolodziej [141] evaluated the efficacy of *L. rhamnosus* GG as a preventative measure for antibiotic-associated diarrhoea in adults and children. The probiotic significantly reduced the probability of AAD, but with higher efficacy in children than in adults. A different study by Evans et al. [142] reported that *L. rhamnosus* R0011 significantly reduced the duration of diarrhoea when compared to antibiotics, in adults with antibiotic-associated diarrhoea. Taking these studies into consideration, *L. rhamnosus* GG offers clinical effects due to its ability to reduce the severity of antibiotic-associated diarrhoea in children [134]. This reduction is attributed to the probiotic’s excellent ability to adhere to the intestinal mucus [13]. Additionally, *L. rhamnosus* has been shown to offer effective prophylactic and therapeutic properties for the treatment of different infections including antibiotic-associated diarrhoea [143,144].

### 4.2. Treatment of Inflammation-Associated Diseases

Modifications of the gastrointestinal microbiome, either by the introduction of pathogens or loss of beneficial microbes, cause allergies and inflammation in immunocompromised individuals [145]. This modification leads to complications such as inflammatory bowel diseases (IBD) that are responsible for increased production of inflammatory cytokines, epithelial cell apoptosis, and immune cell infiltration, leading to disruption of the intestinal epithelial integrity [146]. One of the resultant disorders of inflammation is asthma, which has been reported in 30% of the population worldwide and is characterised by its ability to obstruct airflow and increase the production of mucus and bronchial hyper responsiveness [147]. In the worst-case scenario, asthma can be chronic and affected individuals may suffer considerable morbidity [148,149]. In dairy cattle, inflammation has been associated with uterine diseases such as metritis and endometritis [150]. Uterine inflammation increases the susceptibility of mother cows to *Trueperella pyogenes* infection, which further impairs the reproductive performance of the affected cows [151,152]. These cases have been previously treated with antibiotics; however, there has been extensive concern in public health when these antibiotics are used in animals used as human food [153]. Therefore, there has been a need for an alternative therapy to antibiotics, which could yield a significant positive impact on the dairy industry by reducing economic losses linked to these disorders [154].

Previously, Michail [155] reported that the use of probiotic bacteria resulted in a reduction in allergic airway inflammation. Additionally, several studies reported that the use of probiotics reduced the risk of the development of allergic and inflammatory disorders in humans [66,67,156]. In a study by Yan et al. [157], they reported that two proteins isolated and purified from *L. rhamnosus* (p40 and p75) were responsible for the protection of mice from intestinal inflammation in vivo. The results were in agreement with a study by Wu et al. [158], which revealed that the use of *L. rhamnosus* GG causes a reduction in inflammation in a murine model. In a different study, the presence of *L. rhamnosus* GG reduced apoptosis of primary bovine endometrial epithelial cells [150]. Later, Arnbjerg et al. [145] investigated the effect of *L. rhamnosus* GG on intestinal inflammation and reported a decrease in intestinal inflammation after ingestion of the probiotic. These studies suggest that pre-treatment with the probiotic *L. rhamnosus* could potentially be used as a preventative method in inflammation.

### 4.3. Enhancement of Cholesterol Efflux

Hypercholesterolemia, the presence of high cholesterol in the blood, has been reported as one of the risk factors associated with cardiovascular disease [159]. This relationship was elucidated by Ishimwe et al. [160], who reported that with every 1% reduction in total cholesterol, there is a further 2% decrease in heart disease risk. Atherosclerosis has been described by Yoon et al. [161] as the build-up of lipoprotein cholesterol in the artery wall, which restricts the flow of blood, promoted by inflammation [162]. Studies have reported on the capability of LAB to suppress cholesterol uptake, thus promoting the cholesterol efflux through activation of the liver X receptor, leading to the reduction in whole-body cholesterol levels [63,64]. Kumar et al. [163] stated that probiotics including *Lactobaccilli* and *Bifidobacterium* species could have potential as cholesterol-lowering agents. The consumption of products containing probiotics can decrease elevated human blood cholesterol levels [159], suggesting that probiotics can be used as a good alternative to treating cardiovascular diseases due to their cholesterol-lowering abilities [160].

The cholesterol-lowering capability of probiotics is attributed to their ability to conjugate bile salts [164]. Such deconjugation capabilities have been reported for probiotics such as *L. rhamnosus* BFE 5264 [165]. Yoon et al. [161] investigated the ability of probiotics *L. rhamnosus* BFE5264 and *L. plantarum* NR74 on cholesterol efflux in macrophages. In their study, they found that the probiotics activated the liver X receptor, inducing the cholesterol efflux. Furthermore, Park et al. [166] showed that *L. rhamnosus* BFE5264 inhibits cholesterol build-up; they reported a significant reduction in the levels of cholesterol and atherosclerosis in a mouse model.

## 5. Safety Profile of *L. rhamnosus*

From the definition, it is imperative that the efficacy and safety of all microorganisms need to be verified and thus assessed for whether they still constitute an important part of their characterisation for human use [167]. When studying probiotics, it is crucial to evaluate their safety, characteristics, and specific mechanisms of action to understand their relevance in clinical studies [168]. Previous studies by Salyers et al. [36] and Senok et al. [37] reported that amongst criteria used to select for probiotics, the ability to act as a potential source of antibiotic resistance transfer within the gastrointestinal tract should always be considered. As a safety goal, microorganisms used as probiotics should be beneficial to the host and not exert the risk of antibiotic transfer associated with the normal gut or food microbiota [169,170]. Therefore, it is critical to investigate the antibiotic susceptibility of probiotics as they may not be easily eliminated where they present a negative influence on the host [171,172]. Furthermore, if they carry transferable antibiotic resistance genes, they could be transmitted to other microorganisms [170]. Therefore, it is crucial that when choosing probiotics, care should be taken, and those carrying transferable resistance determinants that can potentially facilitate plasmid transfer should be avoided [173].

Taking that into consideration, undesirable transfer of resistance to endogenous bacteria should be prevented; the probiotics used should not carry resistance other than that required. Some studies have reported on the ability of lactic acid bacteria, including lactobacilli, to act as reservoirs for antibiotic-resistance genes [174]. Tynkkynen et al. [175] investigated whether the vancomycin-resistance genes from *L. rhamnosus* GG can be transferred to a susceptible strain via conjugation. They found that *L. rhmanosus* GG did not contain any plasmids and that it was unable to transfer the vancomycin resistance to enterococcal strains. In addition to this, other studies have shown that in both in vitro and in vivo animal models, as well as in a number of human studies, *L. rhamnosus* GG is a safe probiotic with non-transferable antibiotic resistance [169]. Taking that into consideration, all strains of *L. rhamnosus* used need to be checked for whether they adhere to this safety profile before being used as probiotics.

## 6. Additional Desirable Attributes of *L. rhamnosus*

### 6.1. L. rhamnosus Can Be Incorporated into Varied Delivery Food Vehicles

When incorporated into food products, probiotics need to be able to remain in high enough levels, at least 10^6^ CFU/g of viable cells [176] throughout the storage and during consumption [177]. Health Canada explicitly specifies that bacterial species including *L*. *rhamnosus* must be present at a level of 1 × 10^9^ colony-forming units per serving when delivered in food, while the Italian Ministry of Health regulations require the administration of the same level of viable bacteria in food per day [1]. There is a good body of literature that shows that *L. rhamnosus* can tolerate stresses during manufacturing, specifically when it is incorporated into food products. This ability of *L. rhamnosus* to survive these technological stresses means that it can be easily incorporated into different food products, and consequently increases its accessibility to various consumers. Sunny-Roberts and Knorr [177] investigated the ability of *L. rhamnosus* VTT E-97800 to survive in sucrose-induced osmotic stress. Their results showed that the strain could tolerate the sucrose even at extreme concentrations, suggesting that it can safely be used in sugar-based foods. To confirm this, Cinzia et al. [178] later showed that *L. rhamnosus* was able to survive in jam for up to 78 days when stored at 5 °C. In a different study, Reale et al. [85] investigated the ability of *L. rhamnosus* to withstand different stress factors encountered in food processing. They found that the strains were able to grow in high salt concentrations and at temperatures as high as 55 °C as well as at freezing temperatures. This means that the strain can be investigated for different uses including performance in fermented foods and those stored at fridge conditions. Alamprese et al. [179] showed that *L. rhamnosus* incorporated into ice cream survived for 30 days at −16 °C and up to 1 year at −28 °C. This confirms that *L. rhamnosus* can survive different technological and storage stresses.

Probiotics incorporated into food have a better chance of survival through the gastric passage as they are offered protection by the food vehicles [180,181]. The physical and chemical properties of the food will have a buffering effect and significantly influence the survival of the microorganisms [182]. Food products containing probiotics have been reported to promote health benefits in hosts, and these effects are attributed to characteristics that the viable probiotic strains confer [38,183]. Therefore, there is a need for careful consideration of food matrices that can be used for probiotic incorporation. A wide range of dairy products (milk, ice cream, frozen fermented dairy desserts, yogurt, and cheese) are foods into which probiotics have been added; however, the range of foods has extended to include non-dairy products including meats, confectionary [184,185], and fruit juices [186]. The probiotics need to be active in the food product in order to offer the host beneficial effects [187]. Therefore, it is crucial that the probiotic microbial activity is maintained during the production, packaging, storage, and transportation of these foods. There are a number of studies reporting on the performance of probiotic cultures, such as *Bifidobacterium* and *Lactobacillus*, when incorporated into both dairy and non-dairy products [188,189].

Yoghurt is a fermented milk product resulting from a symbiotic relationship between *Lactobacillus delbruikii* ssp. *bulgaricus* and *Streptococcus thermophilus* under controlled temperature and environmental conditions [190]. Previously, Anukam et al. [191] investigated whether probiotic *L. rhamnosus* GR-1 can be incorporated into yoghurt, survive, and subsequently clear diarrhoea. They found that diarrhoea was resolved in all patients who consumed the yoghurt. Carlsson et al. [192] investigated the feasibility of administering and consuming a drinkable yoghurt containing *L. rhamnosus* LB 21 and *Lactococcus* L1A on the bowel movements and body weight of patients in residential care facilities. They reported that there were no cases of constipation reported from the group that received the yoghurt with probiotics. In a study by Innocente et al. [190], *L. rhamnosus* was added into yoghurt, and its changes in viability during refrigerated storage were monitored. They reported the presence of *L. rhamnosus* at viability values of approximately 7 log CFU/mL after storage, levels which satisfy the minimum recommended level to ensure the potential health promoting effects. With these, yoghurt can easily be used as a vehicle of choice for the probiotic *L. rhamnosus*.

Cheese is considered a convenient alternative vehicle for delivering viable bacteria [193]. Researchers have investigated the incorporation of different probiotics into cheese [194,195]; among those, *L. rhamnosus* has been reported to survive well throughout storage [196]. Boylston et al. [197] reported that *L. rhamnosus* remained viable in different cheeses, without negatively affecting product quality. Cichosz et al. [198] compared the viability of *L. rhamnosus* in cheese-like products and Swiss- and Dutch-type cheese and reported an increase in viable counts to numbers exceeding 8 log CFU/g after ripening. Liu et al. [199] exploited the impact of *L. rhamnosus* on the antioxidant activity of cheddar cheese during ripening and simulated gastrointestinal digestion. They found that there was an enhanced proteolytic activity and antioxidant activity during the ripening process as well as simulated gastrointestinal digestion, signifying the beneficial effects of the probiotic in vitro. When Ningtyas et al. [200] incorporated *L. rhamnosus* into cream cheese, they reported that it remained viable throughout storage without noticeable negative effects, as the pH, moisture, protein, or fat content of the cheese remained the same as those of the cheese without *L. rhamnosus*. These studies have shown that *L. rhamnosus* is able to survive the production of cheese to possibly consequently still offer beneficial effects to the host.

An increase in the demand for healthier foods by the consumer has resulted in the development of even more foods that carry ingredients that will exert beneficial effects [201]. Understanding the importance of the fermentation process and the addition of probiotics to such foods offers an alternative carrier that will be accessible to the greater population. The incorporation of probiotics into most fermented traditional foods in Africa has been reported to offer a more viable opportunity to fight the increasing cases of hunger and malnutrition [202]. Salimei et al. [203] evaluated the effects of incorporating the probiotic *L. rhamnosus* into the maize and sorghum ensiling. They reported that the probiotic showed good survival and improved the fermentation quality, suggesting that there was an increase in the quality of the product. In a different study, Matejčeková et al. [204] investigated the incorporation of *L. rhamnosus* into fermented buckwheat. They reported an increase in viable numbers of *L. rhamnosus* during cold storage of buckwheat, which minimised the outgrowth of contaminating bacteria while enhancing the beneficial effects of the beverage. Recently, Wacoo et al. [205] explored incorporation of *L. rhamnosus* into the fermented cereal beverage, kwete. They reported that fermentation resulted in a product that was acceptable by the consumers and was stable for a longer period. What was worth noting in their study was that supplementation with *L. rhamnosus* enhanced the product’s ability to reduce aflatoxin contamination. Aflatoxin contamination is usually a problem in fermented maize products, staple foods in sub-Saharan Africa. In a different study, Mitra and Ghosh [206] investigated the effects of *L. rhamnosus* on kefir quality characteristics. They reported that there were no significant differences observed in the quality characteristics of the kefir with and without *L. rhamnosus*.

Non-dairy food products have also been gaining attention as probiotics food carriers, especially for lactose-intolerant consumers. Researchers have been studying the alternatives and have proposed that beverages based on fruits, vegetables, cereals, and soybeans can be used as new products containing probiotic strains [207]. Fruit juices, specifically, have been tested for their ability to be probiotic carriers, due to their high nutritional value [208,209], high content of the antioxidant ascorbic acid [207], and the fact that they do not contain starter cultures [209,210]. The absence of starter cultures in juice excludes competition, unlike in dairy products, making it a more appropriate probiotic carrier [208]. Several studies reported that *L. rhamnosus* survived in fruit juices. Sheehan et al. [211] investigated the viability of probiotic *L. rhamnosus* GG over 12-week storage; they reported that it was able to remain viable in low pH juice at commercially acceptable levels. Champagne and Gardner [212] studied the ability of two *L*. *rhamnosus* strains, LB11 and LB24, to survive in orange and pineapple juices; they reported that these strains showed good viability in the juices during storage at 4 °C for 80 days. A study by Ying et al. [213] investigated the effects of microencapsulation of *L. rhamnosus* with whey protein isolate when added into apple juice; their results showed that the food matrix provided protection to the probiotic. Champagne et al. [214] investigated the viability of *L. rhamnosus* R0011 in apple juice when stored in the refrigerator over 5 weeks. They reported that *L. rhamnosus* remained viable when stored over a few weeks in the refrigerator in both opened and unopened bottles.

While the survival of the probiotic in the food during storage is important, it is also important to show that while they are incorporated in food matrices, the probiotic will still survive in vitro and in vivo under simulated gastrointestinal conditions [207,215]. Farias et al. [216] investigated the viability of *L. rhamnosus* ATCC 7496 incorporated in passion fruit juice when exposed to gastrointestinal conditions. They reported that the incorporation of *L. rhamnosus* ATCC 7496 into the juice enhanced its resistance and survival under simulated intestinal conditions. In a similar study, Campos et al. [217] investigated the viability of *L. rhamnosus* in fermented pineapple and jussara juice when subjected to in vitro and in vivo simulated gastrointestinal conditions. They reported that the probiotic survived in the juice for a month and was present in high numbers both in vitro and in vivo. These studies show that *L. rhamnosus* can be incorporated into different fermented products to enhance their beneficial effects while maintaining the product quality. The ability to survive in different products indicates that as a probiotic, *L. rhamnosus* will be accessible to consumers with varied dietary preferences.

### 6.2. L. rhamnosus Is Amenable to the Enhancement of Stability Using Microencapsulation

It is evident that there are different factors that influence the growth of probiotics including technological conditions such as pH, temperature, medium composition, and gastrointestinal conditions after ingestion. There are generally reports of poor survival of probiotics in products during storage and subsequently upon exposure to gastrointestinal conditions [218]. What aggravates the problem during the growth and production of probiotics is that liquid cultures or formulations, which are bound to be bulky and also decrease viability of probiotics, especially at room temperatures, are preferable [219]. This resulted in a need for other formulations that would retain and protect the probiotic cultures even at ambient conditions. Different studies on the development of protective matrices for probiotic bacteria have focused on using microencapsulation [220,221]. Microencapsulation is a technique where solid, liquid, and gaseous materials are retained within an encapsulating matrix or membrane [222] in order to enhance their survival and maintain viability [223,224]. This process is predominantly used for extending the storage life of probiotics and converting them into a powder form for ease of use [225].

When probiotics are encapsulated, they are protected from bacteriophages and harsh environments such as freezing and gastric conditions [225]. Furthermore, microencapsulation enhances the effects of probiotics and has been considered a promising technique for their protection [226]. Different microencapsulation techniques are used, including: spray-drying, freeze-drying, fluidised bed-drying for encapsulating the cultures, and converting them into a concentrated powdered form. Spray-drying is a method of microencapsulation that has emerged as a promising alternative in producing different formulations of probiotic cells. This is a cheap, continuous, and fast process, where probiotics encounter several stresses, such as dehydration and high temperatures, next to atomisation, osmotic, and oxidative stress with an end product that consists of individual powder particles containing the probiotics [227]. Freeze- and vacuum-drying (lyophilisation) is another method of encapsulation that is performed by freezing the probiotics at low temperatures in the presence of cryoprotectants [228]. Another method of encapsulation is the emulsion technique, a two-step procedure involving the dispersion of an aqueous phase containing the bacterial cells and polymer suspension into an organic phase that is then hardened by cooling or the addition of gelling agents [229]. The goal of these techniques is to create an environment in which the bacteria will survive during processing and storage and released at appropriate sites in the digestive tract to confer the beneficial effects [223]. 

The growth phase of cultures plays an important role towards obtaining good viability after encapsulation. It has been shown in studies with *L. rhamnosus* GG that stationary phase cells were more tolerant to stresses encountered during spray-drying while cells from the exponential growth phase were more susceptible [230,231]. Industries such as food production, agriculture, and pharmaceuticals have resorted to using these techniques due to the positive results of yields. Broeckx et al. [227] reported that the use of encapsulation in probiotics can increase the stability and shelf life of the finished probiotic-containing product. It is always crucial to test that the properties are still maintained in the probiotic post-microencapsulation. Thus, the viability and functionality of probiotics should always be assessed during and after encapsulation [232]. All probiotics should retain their functional properties, even after microencapsulation.

A previous study by Sohail et al. [208] investigated the effects of alginate and maltodextrin encapsulation on the survival of *L. rhamnosus* GG after long-term storage at low temperatures. This is especially important considering that most probiotics are incorporated in food stored at low temperatures. They reported that the encapsulated probiotic demonstrated an improved stability during storage at 4 °C for 6 months. Improved heat stability and survivability of the alginate microencapsulated *L. rhamnosus* NRRL 442 cells when compared to the free cells was reported by Shaharuddin and Muhamad [233]. In yet another study, Li et al. [234] showed significantly better survival in simulated gastric and intestinal juices for *L. rhamnosus* GG microencapsulated using transglutaminase treated with soy protein than free cells. Song et al. [235] investigated the antibiofilm potential of alginate–chitosan microencapsulated *L. rhamnosus* GG against *Escherichia coli* biofilm. They reported a reduction in the *E. coli* biofilms in the presence of the microencapsulated *L. rhamnosus* GG cells, suggesting that the microencapsulated cells had the potential to inhibit *E. coli* adhesion, and ultimately the infection.

Other studies looked at the specific encapsulation techniques and reported on their subsequent effects on *L. rhamnosus*. Corcoran et al. [230] showed that spray-drying of *L. rhamnosus* GG resulted in enhanced viability of the probiotic. According to Pimentel-González et al. [131], when *L. rhamnosus* was entrapped in the inner water phase of the double emulsion, its survival significantly increased under low pH and bile salt conditions in an in vitro trial compared to the viability and survival of control cells. Lavari et al. [236] investigated the effects of spray-drying on the capacity of *L. rhamnosus* 64 to modulate the gut immune response and reported that spray-dried probiotic enhanced protection against inflammation in vivo. A study by Azizi et al. [237] encapsulated *L. rhamnosus* ATCC 7469 using sesame protein isolate through spray-drying and found that the method protected the resultant strain against gastrointestinal conditions. Lai et al. [238] evaluated the protective effect of flaxseed mucilage on the co-extrusion microencapsulation of *L. rhamnosus* GG and showed an enhanced protective capacity when exposed to the gastrointestinal environment. Recently, Barajas-Alvarez et al. [239] encapsulated *L. rhamnosus* by spray-drying using gum arabic blended with trehalose as wall materials and investigated its viability in simulated gastrointestinal and storage conditions. The resultant probiotic showed enhanced viability under simulated gastrointestinal conditions and extended shelf life without compromising its beneficial health effects. All these studies show that *L. rhamnosus* can be protected from technological and gastrointestinal stresses while maintaining its probiotic status, using microencapsulation.

## 7. Present Studies on Bioengineering of *L. rhamnosus*

**Figure 4:**
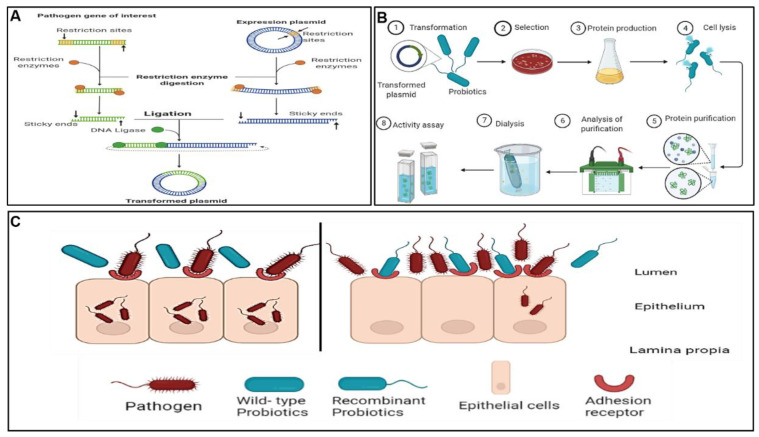
A schematic representation of the steps followed in development of bioengineered probiotics and the mechanism of pathogen inhibition by the bioengineered probiotic strain. (**A**) Cloning and expression of genes of interest from the pathogen into an expression vector. (**B**) Transformation of the expression vector carrying the gene of interest from the pathogen into the probiotic and analyses conducted to confirm expression of the foreign gene by the bioengineered probiotic strain. (**C**) Competitive binding of the bioengineered probiotic strain to the same host receptors to which the target pathogen binds to cause an infection.

A clear understanding of the probiotics’ mode of action as well as the infection cycles of the pathogens inspired the study of recombinant probiotics as an alternative pathogen control and/or treatment agent in the field referred to as pathobiotechnology. Pathobiotechnology is described by Sleator and Hill [240] as the exploitation of pathogenic bacteria, and in particular bacterial virulence strategies, for beneficial applications both in industrial and biomedical applications. In particular, pathobiotechnology can be used to develop robust probiotic strains with attributes such as longer shelf-life stability, enhanced ability to colonise the gut, and marked therapeutic effects, and for the development of unique delivery systems for vaccines [241], drugs [241,242], and therapeutic proteins and gene therapy vectors [242]. An overview of the methodological concepts of bioengineered probiotics was previously reviewed [242]. Mathipa and Thantsha [243] reviewed the various strategies of bioengineering, their successes and limitations, and projections of their possible applications. Figure 4 illustrates the method used for construction of the bioengineered probiotic strain, typical analyses performed to confirm the expression of the foreign gene from the pathogen of interest by the bioengineered strain, as well as the mechanism of competitive inhibition bestowed upon the bioengineered strain by expression of the gene from the target pathogen, which allows it to bind to the same adhesion receptors as the pathogen, thereby enabling the bioengineered strain to better inhibit the target pathogen. This strategy has great potential; however, there have been reports of the dangers associated with using pathogens in any way. Pioneer studies in this field reported successful use of live attenuated pathogens such as *Salmonella*, *Bordetella*, and *Listeria* vaccine vectors, but with the associated potential of the pathogen to revert back to virulent strains, which limits their practical application [244,245,246]. The accidental reversion of live attenuated pathogens has resulted in a need for safer alternatives. To this end, with the increased knowledge of the probiotics mechanisms of action and gene expression, cellular structures can be modified to potentially enhance the beneficial effects and protective functionality of probiotics in the host, making them candidate safer alternatives.

While the idea of using probiotics as treatment for various diseases is not new, engineering them for specific therapeutic applications only became possible in recent years. Using the probiotic engineering strategy, probiotics such as *L. rhamnosus* can be used to confer even more beneficial effects on the host. There have been a number of studies showing the effects of *L. rhamnosus* on pathogens; however, there is little evidence as to the molecular mechanisms by which they interact with and inhibit pathogens. Lebeer et al. [121] investigated whether the pili of the probiotic *L. rhamnosus* GG are key in the adhesion and immunomodulatory factors for intestinal epithelial cells. This was achieved through the production of knockout mutation of the SpaCBA pilus-related genes, a key for efficient adherence to the Caco-2 intestinal epithelial cell line and biofilm formation. In their results, they found that the SpaCBA pili promote strong adhesive interactions with intestinal epithelial cells. A study by Petrova et al. [247] investigated whether lectin-like protein 1 (Llp1) and Llp2 of *L. rhamnosus* GG have antipathogenic properties. They reported that the isolated lectin domains of Llp1 and Llp2 possessed pronounced inhibitory activity against biofilm formation by various pathogens, including clinical *Salmonella* species and uropathogenic *E. coli*. These proteins were then concluded as crucial bioactive ingredients for improved prophylaxis of urogenital and gastrointestinal infections. In a different study, Petrova et al. [248] investigated, through knock-out mutation, the effects of the same lectin-like protein 1 (Llp1) in the adhesion of *L. rhamnosus* GR-1 and subsequent pathogen inhibition. They found that the proteins are responsible for adhesion to the vaginal and ectocervical epithelial cell lines and inhibition of biofilm formation and adhesion of uropathogenic *E. coli.* In an effort to understand the probiotics’ mechanisms of action and their interactions with other microorganisms and as well as the host, Spacova et al. [249] expressed various fluorescent proteins in *L. rhamnosus* GG and *L. rhamnosus* GR-1. This study allowed for the visualisation of adhesion patterns of the recombinant strains to intestinal epithelial cell cultures as well as their inhibition of biofilm formation by pathogens. This manipulation of *L. rhamnosus* allowed for the exploration and better understanding of how it confers the beneficial effects.

The manipulation of probiotics through gene knockout made it possible to understand the mechanisms of action of probiotics; however, the ability of *L. rhamnosus* to inhibit pathogens still remains generic and thus cannot be guaranteed. Having better understood probiotics gene manipulation and the pathogen’s life cycle, there is then an opportunity to engineer strains that will be produced for specific uses. Through probiotic engineering, novel *L. rhamnosus* strains for controlling or treating specific pathogens can be constructed. This can be achieved through cloning and expressing different genes into probiotics to offer alternatives that will confer the probiotics’ beneficial effects along with traits brought upon by the expression of an additional foreign gene or genes (Figure 4). In order to put this approach to the test, Günaydın et al. [250] successfully expressed protein G (GB1-3) in *L. rhamnosus* GG to specifically capture the bovine IgG antibodies and rotavirus. The recombinant strain effectively reduced the prevalence, severity, and duration of diarrhoea in comparison to wild-type *L. rhamnosus* GG. This strain was suggested as a potential alternative to vaccination in individuals where the current vaccines are contraindicated. In a study by Beltran et al. [251], the MAM-7 gene from *Vibro parahaemolyticus* RIMD 2210633 was cloned and expressed in *L. rhamnosus* expression vectors. They reported that the recombinant strain showed enhanced adherence to the Caco-2 epithelial cell line; however, the wild-type strain showed the best capacity to inhibit the pathogen colonisation. A limitation of their study was that although the expression of the MAM-7 gene by *L. rhamnosus* was able to enhance the adherence properties, it did not have the same effects on the capacity of the bioengineered strain to inhibit pathogens. In a different study, Petrova et al. [252] cloned and successfully expressed HIV-inhibiting lectin griffithsin (GRFT) with documented activity against HIV and unknown side-effects on the host cells, into probiotic strains *Lactobacillus rhamnosus* GG and *L. rhamnosus* GR-1. The resultant strains were shown to inhibit T-tropic HIV-1 infection. It was concluded that these recombinant *L. rhamnosus* strains could be used in the inhibition of HIV transmission and replication and ultimately disease progression.

These studies, though not many, show that different researchers are investigating probiotic bioengineering in *L. rhamnosus* for different reasons, either to understand its mechanisms of action or their specific use for different pathogens. The findings from these studies show that recombinant probiotics, specifically *L. rhamnosus*, can play a protective role either in the prevention or control of the colonisation of potential pathogens.

## 8. Conclusions

Due to the increase in the demand for alternative treatment options for pathogens, the use of recombinant *L. rhamnosus* can be studied further. The development of recombinant probiotic strains with the potential to confer dual effects is an interesting avenue. The ability of *L. rhamnosus* to offer enhanced beneficial effects over other probiotics makes it a suitable candidate for the development of recombinant strains. There are a lot of studies looking at the benefits of wild-type *L. rhamnosus* in human hosts; however, studies investigating the benefits of bioengineering of this probiotic are sparse. The successful expression of different specific genes in *L. rhamnosus* will offer dual benefits, conferring probiotic benefits while inhibiting a specific pathogen.

Despite the positive results obtained from the existing studies on bioengineering of *L. rhamnosus*, their limited numbers indicate that more can be accomplished in this field. For example, there are many pathogens for which the *L. rhamnosus* bioengineered strains can be designed for targeted control. Furthermore, studies can look at the incorporation of these recombinant strains into food vehicles so that they are easily accessible to the consumer. The engineered strains can also be formulated using microencapsulation for varied applications. More importantly, the ability of probiotic engineering to enhance their beneficial effects should not result in them being pathogenic in nature. This could be an aspect of further studies for both the existing as well as the new bioengineered strains. The use of probiotics, as opposed to pathogenic strains, as delivery vehicles is currently gaining momentum; thus, using beneficial strains such as *L. rhamnosus* would be a good strategy with a high possibility for the successful development of novel robust probiotics strains with enhanced health benefits.

## Data Availability

Not applicable.
